# SAIF: A Correction-Detection Deep-Learning Architecture for Personal Assistants

**DOI:** 10.3390/s20195577

**Published:** 2020-09-29

**Authors:** Amos Azaria, Keren Nivasch

**Affiliations:** Data Science Center, Ariel University, Ariel 40700, Israel; amos.azaria@ariel.ac.il

**Keywords:** human–agent interaction, correction detection, deep learning, implicit feedback, multimodal architecture

## Abstract

Intelligent agents that can interact with users using natural language are becoming increasingly common. Sometimes an intelligent agent may not correctly understand a user command or may not perform it properly. In such cases, the user might try a second time by giving the agent another, slightly different command. Giving an agent the ability to detect such user corrections might help it fix its own mistakes and avoid making them in the future. In this work, we consider the problem of automatically detecting user corrections using deep learning. We develop a multimodal architecture called SAIF, which detects such user corrections, taking as inputs the user’s voice commands as well as their transcripts. Voice inputs allow SAIF to take advantage of sound cues, such as tone, speed, and word emphasis. In addition to sound cues, our model uses transcripts to determine whether a command is a correction to the previous command. Our model also obtains internal input from the agent, indicating whether the previous command was executed successfully or not. Finally, we release a unique dataset in which users interacted with an intelligent agent assistant, by giving it commands. This dataset includes labels on pairs of consecutive commands, which indicate whether the latter command is in fact a correction of the former command. We show that SAIF outperforms current state-of-the-art methods on this dataset.

## 1. Introduction

Intelligent agents that can interact with users using natural language are becoming increasingly common. Popular operating systems now come with built-in virtual assistants, such as Siri for Apple’s MacOS and iOS, and Cortana for Microsoft’s Windows. As another example, Amazon’s Echo speakers include the Alexa virtual assistant. However, these assistants do not learn from their own mistakes, in contrast to real human assistants.

When humans interact with one another, it often happens that one person misunderstands the other. This person might then realize that she made a mistake by the other person’s reaction. Consequently, she will not only correct her mistake, but she will also learn for the future what the other person’s intentions were in such a situation. For example, when a manager tells her human assistant “I would like to promote Mary”, the assistant might reply “Sure. I sent an email to Mary with the subject ‘You’re promoted’.” Then the manager might reply “I would like to set a meeting to promote her”. The human assistant will then probably recall the email and schedule a meeting with Mary for the promotion. The next time the manager tells the assistant she would like to promote someone, the assistant will remember to set up a promotion meeting.

For personal agents to be truly useful, they should have abilities associated with human intelligence, such as the ability to detect their own mistakes from user reactions. This is an instance of implicit feedback, which is the gathering of information from users’ behavior, as they go along normally using the agent.

A personal agent with the ability to detect user corrections might be able to fix some of the mistakes it makes. For example, suppose a user says “create an email for Tom”, and the agent creates a new email and sets the address to Tom’s address. Then the user says “create an email and set the subject to ‘for Tom’.” The agent might erase the email it created and create a new email in which the subject is set to “For Tom”.

In addition, an agent might learn for the future what a particular user means when giving a certain kind of request. In the above example, if later on the user says “create an email for Nancy”, the agent will create a new email and set the subject to “For Nancy”.

In this paper, we address the problem of detecting an agent’s mistakes by identifying when the user tries to correct the agent. We refer to this problem as the *Correction-Detection* task. We develop an architecture that can detect whether given interactions constitute corrections on the part of the user or not. More precisely, the architecture works on pairs of consecutive commands. We call our architecture *Socially Aware personal assistant Implicit Feedback correction detector* (*SAIF*). It sees only the user’s commands, and not the agent’s responses to those commands, as we would like the architecture to be independent of the agent to which it is applied: A pre-trained version of the architecture should be applicable to any social agent, even though different agents have different responses.

Each pair of consecutive commands can have one of three possible labels: “new command” if the user was satisfied with the agent’s action to the previous command and issued a new command; “command correction” if the user was not satisfied with the agent’s action and tried to correct it; and “ASR correction” if the first command was not carried out properly due to wrong transcription by the Automatic Speech Recognition (ASR) system (for example, “set subject to Johnny” instead of “set subject to join me”).

It is important to separate command corrections from ASR corrections since the actions to be taken by the agent are very different. With an ASR correction, the agent should adjust the ASR component and improve it, so that it does not fail next time. However, when dealing with a command correction, the agent should undo the previous command, and execute the learning process, as it has implicitly learned another way to say the second command.

Our architecture is multimodal, using both the voice (acoustics and non-verbal sounds) as well as the transcript of the user’s spoken commands. This multimodal approach is important, since the voice input can hold important cues such as tone, speed, or emphasis on certain words. Furthermore, voice input can be especially useful in cases where the wrong command was executed due to a fault in the ASR.

### Related Work

Implicit feedback has received a great deal of attention. It encompasses many types of user behavior: the amount of time the user spends seeing a document or a web page, her scrolling and clicking behavior, whether she copies parts of it, creates a bookmark, and so on. Oard and Kim [1] developed an early classification system for types of implicit feedback, based on the type of behavior, as well as based on its scope, which could be part of a document, a whole document, or a whole class of documents. Kelly and Teevan [2] later expanded this classification system. Their paper gives a broad survey of previous work on implicit feedback. Recently, Jannach et al. [3] further updated and expanded this classification system, and gave an updated survey of this area.

Search engines can use implicit feedback, such as clicking behavior, follow-up search queries and even eye-tracking, to improve the ranking of search results. The act of down-ranking one search result and up-ranking another can be considered a correction performed by the search engine in response to the user’s behavior. Implicit Feedback in search engine results often relies on the user choice among the ordered search results. Hence, it differs from the task in this work.

Levitan and Elson [4] described a method for detecting retries of voice search queries. Their task is quite similar to the one in this work, as their recognizer takes as input pairs of consecutive search commands to be classified. However, their recognizer takes as input only the transcripts of the commands. More significantly, their classification system is different, since it is binary and furthermore, if the ASR transcribed correctly, the instance is labeled as “no error”, even if the user subsequently tried to correct the agent.

Zweig [5] proposed some methods for improving the accuracy of ASR translation when the user repeats her search command. In his work, recognition of repetitions is based on the fact that the user did not choose any of the options that were shown to him after his first search command. In contrast, we try to recognize user corrections from the commands themselves. Furthermore, sometimes a correction may not look like a repetition of the previous command.

Heeman and Allen [6] considered the problem of recognizing speech repairs in spoken sentences, which occur when the speaker goes back and changes or repeats something she just said. However, in our case we try to recognize when a complete command is a correction of a previous complete command.

Bechet and Favre [7] aimed to detect errors in ASR output using a combination of ASR confidence scores, and lexical and syntactic features. If the system detects a problem, it requests the user for a clarification. Ogawa and Hori [8] also aimed to detect ASR errors, using deep bidirectional RNNs. In our work, the objective is broader, since we want to detect not only ASR errors, but also user corrections unrelated to the ASR.

Paraphrase detection is the task of deciding whether two given sentences have the same meaning even though they use different words. The Microsoft Research Paraphrase Corpus [9] is a database of labeled pairs of sentences, some of which are paraphrases of one another. There are several works on paraphrase detection based on this corpus.

In particular, Kiros et al. [10] developed an off-the-shelf sentence-to-vector encoder called Skip-Thoughts, which they applied to paraphrase detection, as well as to several other learning tasks. Skip-Thoughts tries to reconstruct the surrounding sentences of an encoded passage, using the continuity of the training text. Sentences that share semantic and syntactic properties are thus mapped to similar vector representations. Skip-Thoughts also includes a vocabulary expansion method to encode words that were not seen as part of training.

Agarwal et al. [11] developed a paraphrase detection method that works well with short noisy data such as Twitter texts. See also [10,12,13,14,15,16,17].

Paraphrase detection is closely related to our Correction-Detection problem. Indeed, a user might try to correct an agent by repeating the previous command in slightly different words. For example, the user might give the command “remove the contact Tom” and the agent might not understand or not perform it correctly. The user might try again in different words by saying “delete the contact named Tom”.

However, there are several differences between paraphrase detection and the Correction-Detection task. The second command might constitute a correction of the first, even though it has a slightly different meaning: The two commands might differ in proper names (e.g., Tom vs. John) or in numerical quantities, and the user’s tone of voice might indicate that he got confused in the first command. Furthermore, in our task the order of the commands might be significant. For example, the agent might understand the word “create” but not the word “compose”. Hence, the order between the commands “create an email for Tom” and “compose an email for Tom” is very significant.

Another similar task is the Quora Question Pairs competition, which challenges participants to tackle the problem of identifying duplicate questions [18]. Choudhary addressed this problem using BERT [19] (See also [20,21]).

Multimodal deep learning has been applied to tasks such as speech recognition, speech synthesis, emotion and affect detection, media description, and multimedia retrieval [22,23,24,25,26,27]. To the best of our knowledge this is the first research on multimodal voice and transcript deep learning for Correction Detection.

## 2. Materials and Methods

### 2.1. Formal Problem Definition

Assume a dataset of size *n* coming from multiple users interacting with a personal assistant agent. Let $C=\{c_{1},c_{2},\ldots ,c_{n}\}$ be a set of commands given to a personal agent. Each of the commands, *c*, is composed of a transcript of the command, $c^{t}$, the command voice, $c^{v}$, and an indicator of the agent’s success in executing the command, $c^{s}$. Let $t(c_{i},c_{j})$ be a function that associates commands $c_{i}$ and $c_{j}$ with a type in {new,asr,cc}, where new denotes no relation between two commands (that is, the $c_{j}$ is a new command), asr denotes that $c_{j}$ was given in order to correct a malperformance of the transcription performed by the agent, and cc denotes that $c_{j}$ is an attempt of the user to refine and correct $c_{i}$.

In this paper, we focus on the consecutive multimodal correction-detection problem, in which for each command $c_{i}$, the value of $t(c_{i},c_{i+1})$ must be determined.

### 2.2. Dataset Description

To develop our architecture, we use a set of real interactions that users had while experimenting with the social agent *LIA* (*Learning by Instruction Agent*) [28,29]. This dataset contains a series of 2540 pairs of spoken commands given to LIA by 20 different users, of which 11 were male and 9 were female. The users’ ages ranged from 18 to 62, with a mean of 36.9. For each command we have the original voice file and the written transcript produced by the ASR. The average command consists of 3.6 words and it lasts 4.2 seconds. Each command is followed by a response from the agent.

We manually labeled each pair of consecutive commands according to whether the second one is a correction of the first. As we mentioned, there are three possible labels: no correction (“new command”), a correction in which the user provides a different command (“command correction”), and correction due to incorrect ASR transcription (“ASR correction”). At first we tried to have the labeling done through the Mechanical Turk. However, we got very poor results, so we had to perform the labeling ourselves. Out of the 2540 commands, 568 commands are labeled as a “command correction”, 236 of them are labeled as an “ASR correction”, and the rest are as “new command”. See Table 1 for some examples.

When labeling each command, we relied on the previous command as well as on the agent’s response to it to decide whether the command is a correction (even though as we mentioned, the architecture sees only the commands themselves but not the agent’s responses). We also have an indicator from LIA that specifies whether the command was executed successfully or not. The dataset is available at [30].

### 2.3. SAIF Architecture

To address the correction-detection problem, we developed a multimodal architecture, SAIF. SAIF uses both voice and transcript inputs. Each input instance (*c*) consists of the voice ($c^{v}$) and transcripts ($c^{t}$) of two consecutive commands ($c_{i},c_{i+1}$).

**Figure 1:**
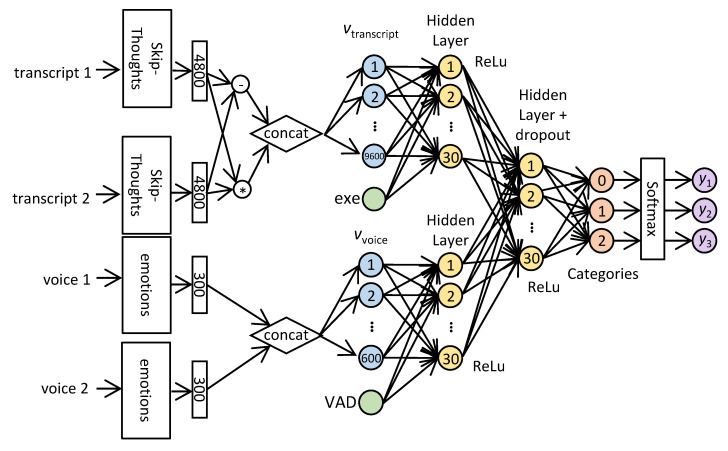
SAIF Architecture.

SAIF first converts the inputs to vector representations and encodes each command transcript ($c_{i}^{t}$) as a vector $s_{i}$ of length 4800 using the Skip-Thoughts encoder [10] (see Section 2.4 below). SAIF then computes the component-wise product and the absolute difference of these two vectors and concatenates the results, obtaining a single vector $v_{\mathrm{transcript}}$ of length 9600, i.e., SAIF computes $v_{\mathrm{transcript}}=(s_{i}\circ s_{i+1},|s_{i}-s_{i+1}\left|\right)$. To this vector, SAIF appends the feature $c_{i}^{s}$ (marked as *exe* in Figure 1), which indicates whether the agent executed the first command or not, resulting in a vector $v_{\mathrm{transcript}}^{\prime }$ of length 9601.

Additionally, SAIF converts the voice commands ($c_{i}^{v}$) into vectors. For this, it uses a model from DataFlair [23] for emotion recognition (see Section 2.4 below). Using this pre-trained model, SAIF encodes each voice file into a vector of length 300. SAIF then concatenates the encodings of the two voice commands, obtaining a vector $v_{\mathrm{voice}}$ of length 600. To this vector, SAIF appends a feature *VAD* related to voice activity detection: Using the WebRTC library [31], SAIF measures the length $\ell_{i}$ of the portion within each sound command $c_{i}^{v}$ which constitutes actual speech. The feature *VAD* equals the difference $\ell_{i+1}-\ell_{i}$. Denote the resulting vector of length 601 by $v_{\mathrm{voice}}^{\prime }$.

The vector $v_{\mathrm{transcript}}^{\prime }$ is then fully connected to a Hidden Layer $H_{1}$ of 30 neurons and ReLu activation. Similarly, the vector $v_{\mathrm{voice}}^{\prime }$ is fully connected to another Hidden Layer $H_{2}$ of 30 neurons and ReLu activation. This vector of length 60 is then fully connected to a third Hidden Layer $H_{3}$ of 30 neurons with dropout of 0.5 and ReLu activation.

The output of $H_{3}$ is linearly fully connected to a layer of size 3 which corresponds to the three possible label values. Finally, we apply SoftMax on this layer, resulting in a vector with three probabilities. The architecture is illustrated in Figure 1.

### 2.4. Pre-Training Methodologies

SAIF uses pre-trained models for encoding both the transcript and voice inputs. Pre-trained models enable transfer of learning and can boost accuracy without taking much time to converge, as compared to training a model from scratch.

The model used for encoding the transcripts is Skip-Thoughts by Kiros et al. [10]. This model is trained on the BookCorpus dataset which is a large collection of novels written by yet unpublished authors. The dataset has books in 16 different genres, e.g., Romance (2865 books), Fantasy (1479), Science fiction (786), Teen (430), etc. Altogether, it contains more than 74 million sentences. Along with narratives, books contain dialogue, emotion and a wide range of interaction between characters. With a large enough collection, the training set is not biased towards any particular domain or application.

Kiros et al. then expand their model’s vocabulary by learning a linear mapping from a word in word2vec space to a word in the encoder’s vocabulary space. The mapping is learned by using all words that are shared between vocabularies. After training, any word that appears in word2vec can then get a vector in the encoder word embedding space. Thus, even though their model was trained with only 20,000 words, after vocabulary expansion it can successfully encode almost one million possible words.

The model used for encoding the voice inputs is based on the emotion recognition model by DataFlair [23] which is pre-trained on the RAVDESS database [25] and uses a multi-layer perceptron (MLP) classifier. The RAVDESS database contains 7356 voice files from 24 actors, rated by 247 individuals 10 times on emotional validity, intensity, and genuineness. The files are labeled into eight different types of emotions (neutral, calm, happy, sad, angry, fearful, disgust, surprised). SAIF takes the last activation layer of this model to obtain a vector of size 300. The entire dataset is 24.8 GB.

## 3. Results

SAIF was trained and tested on the dataset mentioned in Section 2.2, as follows: An array containing all the input instances (each of which contains the voice and transcripts of two consecutive commands) was created and randomly shuffled. A 5-fold cross validation was performed: Five rounds were run, where in each round, 2032 input instances were used as training data and 508 input instances were used as test data. The training used minibatches of size 128, employing TensorFlow’s Adam algorithm for optimization with a learning rate of 0.001. The training loop ran for 10000 iterations or until the train accuracy exceeded 0.995. Hence, each input instance belonged once to the test data. After averaging the results of the five tests, the obtained average test accuracy was 0.818. Since the “new command” instances constitute 68% of the data, guessing all the time “new command” would yield an accuracy of only 0.68. The SAIF code is available at [30]. Table 2 shows the confusion matrix of the results. As shown in the table, SAIF is correct most of the time.

In addition, Table 3 shows two groups of baselines. The first group shows some transcript-only approaches while the second group shows some voice-only approaches.

We modified SAIF to use only voice inputs or only transcripts. In these cases, the accuracy and F1 measures decreased, showing the importance of the multimodal approach. The “transcript+exe” architecture gave an accuracy slightly lower than SAIF. However, the F1 measures were noticeably lower, in particular the F1 measure of “ASR correction”.

In the first group of baselines, we show the result given by the Skip-Thoughts paraphrase detection code of [10], which was slightly modified to match our methodology. We also tried replacing Skip-Thoughts by BERT [32] in two different ways. We first tried using BERT as a text encoder, encoding each sentence separately. We also tried entering the transcript pairs in parallel following the BERT-based architecture of Choudhary [19]. In both cases, we got worse results. See Table 3.

In the second group of baselines, we show the result given by the Dynamic Time Warping (DTW) method [33], which measures the similarity between the two voice commands; these values then served as an input to a neural network.

We note that the Skip-Thoughts baseline method results in an accuracy of 0.742 only. Moreover, it correctly predicted only a very small number of ASR corrections. This deficiency is reflected in the very low F1 score for the “ASR correction” label. The voice-based architectures (“voice+VAD” and “voice only”) gave very poor results, and so did the DTW baseline. These three architectures guessed “new command” almost exclusively.

Clearly, SAIF achieved the best results. Among the three voice-based architectures that were tested, the “voice+VAD” slightly outperformed the other two voice-based methods, especially in detecting ASR corrections. We note that adding the voice features to the transcript features seems to help mostly in detecting ASR corrections, but also the command correction F1 slightly improves.

### Discussion

As stated, the correction-detection problem is different from paraphrase detection. One difference is reflected in the fact that the order of the sentences is significant. To highlight this difference, we ran another evaluation in which we switched the order of the inputs during the test phase. This act decreased the accuracy to 0.713.

The voice component of the architecture relies on a model that is pre-trained on the RAVDESS database, which contains 7356 voice files. For comparison, the Skip-Thoughts model, which we used for the transcripts, is pre-trained on more than 74 million sentences. We believe that using a larger voice database for the pre-training will produce better voice features, which will improve the performance of the voice part of SAIF.

It might be possible to improve SAIF’s performance by making it look at three or more consecutive commands, instead of only two. For example, if the user says “set the subject to hello” and the agent responds that it does not know to which email to set the subject, then the user might try to correct the agent using two further commands: “create new mail”, and “set the subject to hello”. In cases like these, SAIF would be in a much better position if it had access to all three commands. If we refer back to the formal definition of the correction-detection problem (See Section 2.1), in the more general correction-detection problem, $t(c_{i},c_{j})$ must be determined for every i<j and is no longer limited to j=i+1 (as it is in the consecutive correction-detection problem). Furthermore, we may define $t(c_{i},S)$ as a function that determines for every set (or sequence) of commands S⊂C whether it is a correction of the command $c_{i}$, and, if so, what type of correction it is. It may also be possible to improve the ASR performance using the techniques of Bechet and Favre [7] and Ogawa and Hori [8], and in case of repeated utterances by using also the techniques of Zweig [5].

## 4. Conclusions and Future Work

In this paper, we considered the problem of automatically detecting user corrections using deep learning based on multimodal cues, i.e., text and speech. We developed a multimodal architecture (SAIF) that detects such user corrections, which takes as inputs the user’s voice commands as well as their transcripts. Voice inputs allow SAIF to take advantage of sound cues, such as tone, speed, and word emphasis. We released a labeled dataset of 2540 pairs of spoken commands that users had with a social agent. The dataset includes three types of labels: “new command”, “command correction”, and “ASR correction”. We ran SAIF on the dataset; SAIF achieved an accuracy of 0.818 and F1 measures of 0.69, 0.344 for the “command correction” and “ASR correction” labels, respectively. We showed that SAIF outperforms several other architectures, including architectures based on BERT. We believe that releasing the dataset will lead to further work on this problem.

The multimodal correction-detection problem presented in this work has many implications to social interactive agents and personal assistants. Therefore, in future work we intend to assemble SAIF in a personal agent, and use the implicit feedback obtained by correction detection to learn aliases to commands and to undo commands that were unintentionally given by the user. However, SAIF must be adjusted so that it has very high precision for the agent to be effective. High precision is required since undoing commands that the user did not intend to undo, or learning incorrect aliases, may impair the use of the agent. Assuming a high precision, the agent can learn from the examples marked as command corrections, even if the recall is relatively low. Alternatively, when suspected, the agent may explicitly ask the user whether a given command is indeed a correction, or, treat a command as a correction only if it appears as a correction more than once, or by more than a single user.

## Figures and Tables

**Table 1:** Dataset examples.

Command 1	Agent Response	Command 2	Label
Create a new email	Composing new email. “outgoing email” fields are: subject, body and recipient list. What shall I do next (when executing: “tell Tom to buy milk”)?	set the recipient to Tom’s email address	“new command”
Create a contact	Concept “contact” was defined successfully. Please add fields to it.	a contact has an email address	“new command”
I’m running late	Sorry, but I don’t understand. Would you like to teach me (say yes or just ignore)?	tell Tom I’m running late	“command correction”
How long can a whale stay underwater	Sorry, but no answer was found.	how long can whale wait underwater	“command correction”
Play the email	execute command {playYouTube}	read email	“command correction”
Who was the present of the United States	Tuesday, 20 June 2017	who was the president of the United States	“ASR correction”
In for Mariam late	Sorry, but I don’t understand. Would you like to teach me (say yes or just ignore)?	inform Mary I’m late	“ASR correction”

**Table 2:** Confusion matrix of SAIF test results.

Actual Values	Predicted Values		
	New Command	Command Correction	ASR Correction
New command	**1637**	71	28
Command correction	151	**378**	39
ASR correction	95	78	**63**

**Table 3:** Comparison between different experiments.

	Accuracy	Command Correction F1	ASR Correction F1
**SAIF (Multimodal)**	**0.818**	**0.69**	**0.344**
transcript+exe	0.805	0.678	0.255
transcript only	0.755	0.575	0.212
Skip-Thoughts	0.742	0.563	0.076
BERT (encoder)	0.73	0.564	0.186
BERT (2-parallel)	0.709	0.497	0.335
voice+VAD	0.68	0.03	0.047
voice only	0.677	0.006	0.015
DTW	0.681	0.012	0
